# Modification of Graphitic Carbon Nitride with Hydrogen Peroxide

**DOI:** 10.3390/nano10091747

**Published:** 2020-09-03

**Authors:** Petr Praus, Aneta Smýkalová, Kryštof Foniok, Vlastimil Matějka

**Affiliations:** 1 Department of Chemistry, VŠB-Technical University of Ostrava, 70800 Ostrava, Czech Republic; aneta.smykalova@vsb.cz (A.S.); krystof.foniok@vsb.cz (K.F.); vlastimil.matejka@vsb.cz (V.M.); 2 Institute of Environmental Technology, VŠB-Technical University of Ostrava, 70800 Ostrava, Czech Republic

**Keywords:** graphitic carbon nitride, hydrogen peroxide, modification, characterization

## Abstract

Graphitic carbon nitride (GCN) was synthetized by heating melamine and then it was thermally exfoliated for 1–3 h in air. Both bulk and exfoliated GCN nanomaterials were treated in the 10–30% aqueous solutions of H_2_O_2_ for us to study their modification. The light absorption properties were observed by the reddish color and the red-shifts of their UV-Vis spectra. The content of oxygen increased and hydrogen peroxide was supposed to partially oxidize C-OH groups to C=O ones and to form C-O-C groups instead of edge C-NH-C ones. The GCN structure changes were not observed. However, a surface modification of the GCN materials was recognized by their changed photocatalytic activities tested by means of Acid Orange 7 (AO7) and Rhodamines B (RhB), zeta-potentials, and neutralization titration curves.

## 1. Introduction

Graphitic carbon nitride has been intensively studied, especially since 2009 when a paper about its photocatalytic properties was published by Wang et al. [1]. It is a 2D semiconducting nanomaterial with interesting properties, such as high thermal, physical, chemical and photochemical stability [2,3]. It absorbs visible irradiation as a result of the band gap energy of 2.7 eV. This nanomaterial is potential for a lot of applications, such as solar cells manufacturing [4], imaging, sensing of some compounds [5,6,7,8,9], etc. In addition, the special interest has been concentrated on its catalytic [10] and photocatalytic utilization [11,12,13,14,15,16]. However, there are some drawbacks associated with graphitic carbon nitride (GCN), such as fast recombination of photoinduced electrons and holes and low specific surface area.

The doping of GCN with metals and non-metals enables tuning its band gap energy to enhance the absorption of visible light, facilitates the separation of photoinduced electrons and holes and improves other physico-chemical properties [17,18,19,20,21]. Especially, attention has been paid to its doping with environmental-friendly non-metals, such as S, O, P, and N.

One of the procedures used for the GCN doping with oxygen is the formation of oxygen functional groups by the treatment of already prepared GCN with H_2_O_2_ [22,23,24,25,26,27,28,29]. Hydrogen peroxide was also hypothesized to create some defects in the GCN structure [25]. The treatment with H_2_O_2_ is easy but interpretation of the results is not unambiguous. Oxygen functional groups, such as N-O [22] and N-C-O [22,24,25,26], were supposed to be formed in the GCN structure. Other authors observed the formation of C-O-C [23] and C-OH groups [23,27] as well. Therefore, the aim of our work was to verify if H_2_O_2_ is suitable for the doping of already synthetized GCN with oxygen and how the GCN physico-chemical properties can be changed.

The bare and treated GCN nanomaterials with H_2_O_2_ were studied by means of common solid-state characterization methods, such as UV-Vis diffuse reflectance (DR) spectroscopy, photoluminescence (PL) spectroscopy, Fourier transform infrared (FTIR) spectroscopy, X-ray diffraction (XRD), X-ray photoelectron spectrometry (XPS), transmission electron microscopy (TEM) and scanning electron (SEM) and transmission electron microscopy (TEM), physisorption of nitrogen, and measurement of zeta potentials. The content of H, C, N was determined by elemental analysis (EA) and the content of oxygen was calculated up to 100%. Their photocatalytic activity was tested by the decomposition of Acid Orange 7 (AO7) and Rhodamines B (RhB).

## 2. Materials and Methods

### 2.1. Chemicals

All chemicals used were of analytical-reagent grade. Melamine, Acid Orange 7, and Rhodamine B were obtained from Sigma-Aldrich (Darmstadt, Germany). NaOH and HCl were purchased from Lach-ner (Neratovice, Czech Republic). Distilled water was used for the preparation of all solutions and experiments.

### 2.2. Preparation of Bulk and Exfoliated GCN

Bulk GCN was prepared in air by heating of melamine at 550 °C for 4 h with the heating rate of 3 °C min^−1^ in a ceramic crucible with a lid (diameter 5 cm, 30 mL) in a muffle furnace. The crucible was cooled down to room temperature out of the furnace and then grounded in an agate mortar into a fine powder.

The GCN exfoliation was also carried out in air by heating of the bulk GCN in a thin layer on a ceramic plate (diameter 8 cm, 50 mL) at 500 °C in the muffle furnace for 1–3 h with the heating rate of 10 °C min^−1^. The ceramic plate with the product was cooled down to room temperature out of the furnace. The bulk GCN was labelled as Bulk and the GCN exfoliated for 1, 2, and 3 h were labelled as Ex1, Ex2, and Ex3.

### 2.3. Treatment of GCN Nanomaterials with Hydrogen Peroxide

One gram of a GCN sample was placed in a 100 mL glass autoclave together with 50 mL of the 10%, 20%, and 30% hydrogen peroxide solution and then mixed for 5 min. After mixing, the autoclave was put into an oven at 150 °C for 5 h. The resulting dark yellow nanomaterial was filtered, washed several times with deionized water, and dried overnight at 105 °C.

### 2.4. Elemental Analysis

The analysis of C, N, and H in the GCN nanomaterials was performed by means of a CHNS 628 analyser (Leco Corporation, St. Joseph, MI, USA). The samples (100 mg) were burned in oxygen atmosphere at 950 °C and product oxides were registered in an IR cells. The measurement range was of 0.2–60%. The content of oxygen was calculated up to 100%. The relative error of EA was 3–5%.

### 2.5. UV-Vis Spectrometry

The UV-Vis DR spectra were registered using a spectrophotometer Shimadzu UV-2600 (IRS-2600Plus, Shimadzu, Kjóto, Japan) at room temperature. Measured diffuse reflectance was transformed to Schuster-Kubelka-Munk’s function as follows

(1) $$F\left(R_{\infty }\right)=\frac{{\left(1-R_{\infty }\right)}^{2}}{2R_{\infty }}$$

where *R*_∞_ is the diffuse reflectance of a semi-infinite layer.

### 2.6. Photoluminescence Spectrometry

The PL spectra were recorded using a spectrometer FLS920 (Edinburgh Instrument Ltd., Edinburgh, UK). The spectrometer was equipped with a 450 W Xenon lamp (Xe900). The excitation wavelength was 325 nm. The width of excitation and emission slits was 3 nm.

### 2.7. FTIR Spectrometry

The FTIR spectra were recorded using a Nicolet iS50 device (Thermo Scientific, Waltham, MA, USA) and the KBr pellet technique was employed. A small amount of sample was mixed and homogenised with KBr (approximately 200 mg) and pressed at a pressure of 20 MPa to obtain a transparent tablet. The prepared sample was placed in a holder of a transmission attachment where spectra were collected in the wavenumber range of 500–4000 cm^−1^ with the resolution of 2 cm^−1^.

### 2.8. X-Ray Diffraction Analysis

The XRD patterns were recorded by means of a Rigaku SmartLab diffractometer (Rigaku, Tokyo, Japan) with a detector D/teX Ultra 250. A source of X-ray irradiation was a Co tube (CoKα, λ_1_ = 0.178892 nm, λ_2_ = 0.179278 nm) operated at 40 kV and 40 mA. The XRD patterns were recorded between 5° and 90° of 2θ with the step size of 0.01° and speed 0.5 deg min^−1^. The crystallite size *L* was evaluated using Scherrer’s equation for broadening *B*(2θ) (in radians) at a half maximum intensity (FWHM) of a diffraction band as follows

(2) $$B\left(2\Theta \right)=\frac{K\lambda }{Lcos\Theta }$$

where *λ* is the wavelength of X-rays, θ is Bragg’s angle and the constant *K* was set at 0.9.

### 2.9. XPS Analysis

The XPS analysis was performed by means of an X-ray Photoelectron Spectrometer ESCA 3400 (Kratos Analytical, Manchester, UK) with a base pressure in the analysis chamber of ~5.0 × 10^−7^ Pa. Electrons were excited with an Mg Kα radiation generated at 12 kV and 10 mA. For all spectra, the Shirley background was subtracted. Peaks in spectra concerning the sp^2^ hybridized nitrogen (C=N-C) were set to 398.8 eV as a charge correction.

### 2.10. Physisorption of Nitrogen

The physisorption of nitrogen was measured at −196 °C using a device SORPTOMATIC 1990 series (Thermo Scientific, Waltham, MA, USA). The obtained data were evaluated by means of the Brunauer–Emmett–Teller (BET) method.

### 2.11. SEM Analysis

Microscopic investigations were performed using a scanning electron microscope (FEI Quanta 450, Thermo Fischer Scientific, Waltham, MA, USA) with a field emission gun and energy-dispersive X-ray spectroscopy (EDAX). The SEM micrographs were obtained using secondary electrons (SE) with an acceleration voltage of 20 kV and spot size 5. The samples were applied on SEM pins by the use of a carbon conductive double-tape with Al interlayer.

### 2.12. TEM Analysis

Transmission electron microscopy was performed by means of a JEOL 2100 microscope with a LaB6 electron gun. The accelerating voltage of 160 kV was applied. Micrographs were taken by a camera Tengra (EMSIS). The samples were prepared by dispersion in ethanol and sonication for 5 min. One drop of this solution was placed on a copper grid with a holey carbon film and dried at room temperature.

### 2.13. Photocatalytic Experiments

The photocatalytic activity of the GCN nanomaterials was investigated by means of the decomposition of AO7 and RhB in the concentration of 25 mg·L^−1^ and 10 mg·L^−1^, respectively. In dark, into 150 mL of this solution 45 mg of each nanomaterial was added and stirred for 60 min to reach adsorption-desorption equilibria. Then, the suspension was irradiated with a LED source (420 nm). The samples of 2 mL were taken and absorbances at 485 nm for AO7 and 496 nm and 554 nm for RhB were measured using a UV-Vis spectrometer Helios (Thermo Scientific, Waltham, MA, USA). Their full spectra were recorded by a spectrometer Shimadzu UV-2600 (IRS-2600Plus, Shimadzu, Kjóto, Japan).

### 2.14. Statistic Calculations

Statistical calculations were performed using the software packages QC.Expert (Trilobyte, Pardubice, Czech Republic) and XLSTAT 2018 (Addinsoft, Boston, MA, USA) at the α = 0.05 significance level.

## 3. Results and Discussion

The bulk GCN was synthetized from melamine in air and then it was exfoliated for 1, 2 and 3 h. These nanomaterials were treated with the 10%, 20%, and 30% solutions of hydrogen peroxide. The physico-chemical properties of all the GCN nanomaterials were studied using several solid-state characterization methods including photocatalysis.

### 3.1. Elemental Analysis

The elemental analysis was performed for us to find the content of C, N, H and O in the GCN nanomaterials, see Table 1. The C/N ratios between 0.560 and 0.568 indicate that some insignificant structure changes occurred. However, the contents of oxygen after the treatment with H_2_O_2_ were mostly higher than those before the treatment. It implies that hydrogen peroxide brought more or less additional oxygen into the GCN structures. The content of carbon and nitrogen can be reduced by decarboxylation [30] and the removal of amino groups [31], respectively, as a result of the treatment.

The formation of hydroxyl groups during their synthesis and exfoliation was explained by disclosing of double bonds -C=N- to -C(OH)-NH- [32]. Their oxidation to -C=O groups can be supposed as well. The carbonyl groups can act as chromophores causing the red-shifts of UV-Vis absorption spectra, as discussed below. The similar effect of -C=O groups on the band gap energies was referred by Yang et al. [31]. However, the oxidation of –COH to C=O does not explain the increased amount of oxygen which was further investigated.

### 3.2. UV-Vis Spectrometry

**Figure 1:**
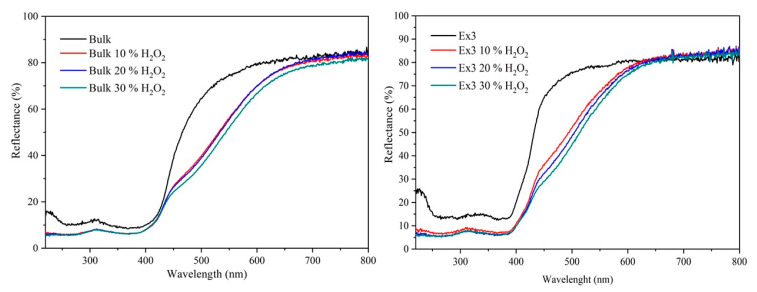
UV-Vis diffuse reflectance spectra of bulk (**left**) and exfoliated GCN nanomaterials (**right**).

The UV-Vis spectra demonstrated in Figure 1 were registered for us to observe the light absorption properties of the GCN nanomaterials. The reflectance decrease, which means the increase of light absorption, is remarkable for the GCN nanomaterials treated with H_2_O_2_. Additionally, their red-shifts were registered which can be attributed to the role of the created chromophores already mentioned. The photographs of these nanomaterials are displayed in Supplementary nanomaterials in Figures S1 and S2.

The optical band gap energies (hereinafter the band gap energy) were evaluated by plotting (F(R_∞_)·hν)^1/2^ against hν and then by means of the well-known Tauc´s procedure [33] considering indirect electron transition [34,35,36,37,38]. The evaluated band gap energies (E_g_) are summarized in Table 2.

The *E_g_* values of the GCN nanomaterials after the treatment were always lower than those before the treatment. It confirms the red-shits of light absorption mentioned above. The *E_g_* increase due to exfoliation is not surprising and was already observed, e.g., [39]. It was documented by the increase of SSA given in Table 2. However, these data show that there is no significant correlation (Spearman *r* = 0.427) between the SSA and the content of oxygen which means that hydrogen peroxide did not affect the GCN structure. It was also observed by XRD, SEM, and TEM, as discussed below.

### 3.3. Photoluminescence Spectrometry

**Figure 2:**
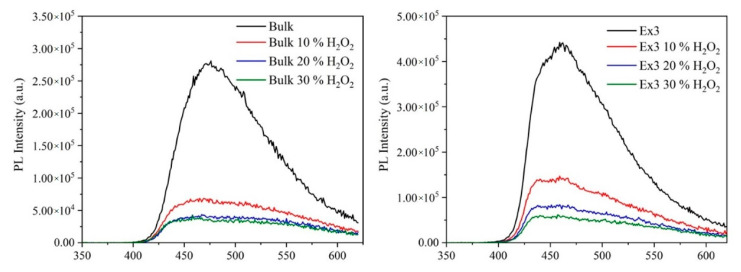
Photoluminescence spectra of bulk (**left**) and exfoliated GCN nanomaterials (**right**).

The PL spectra of the GCN nanomaterials are displayed in Figure 2. GCN is known to have delocalized electrons in its conjugated system, forming π* antibonding orbitals. Therefore, the PL spectra are mostly caused by the electron-hole transmission between π* conduction bands and lone pair valence bands [40].

The photoluminescence intensity decrease corresponding to the treated GCN nanomaterials is remarkable. It is also obvious that when the higher concentration of H_2_O_2_ was used the lower PL was observed likely due to absorption of PL irradiation by the created chromophores. This is another indication that hydrogen peroxide modified the bulk and exfoliated GCNs.

### 3.4. X-Ray Diffraction Analysis

**Figure 3:**
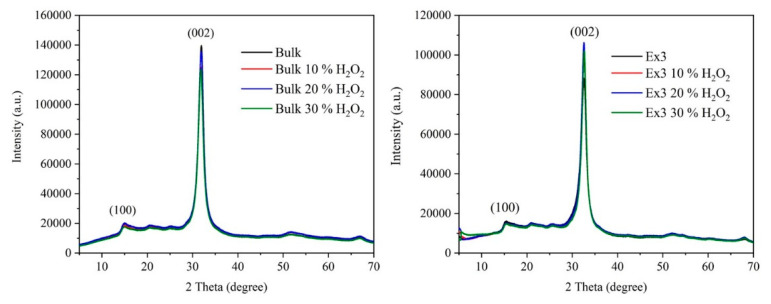
X-ray diffraction (XRD) patterns of bulk (**left**) and exfoliated nanomaterials (**right**).

The structure of the bare and treated GCNs was studied by XRD and only some selected diffractograms are presented in Figure 3. Two typical diffraction peaks at about 15° and 31–33° 2Θ correspond to the (100) and (002) diffractions. They are attributed to the hexagonal structure of graphitic carbon nitride (JCPDS 87-1526). The more intensive (002) diffraction peak corresponds to interlayer stacking of the (002) melem planes. The less intensive (100) one is attributed to the in-plane arrangement of nitrogen-linked heptazine units [41]. Some XRD characteristics are given in Table 3. The values of the interlayer distance *d*(002) were typical for CGN and can be found anywhere in the literature.

The crystallite sizes were tested for their normality which was confirmed by the selected statistical tests: Shapiro-Wil (*p* = 0.290), Anderson-Darling (*p* = 0.206), Lilliefors (*p* = 0.420), Jarque-Bera (*p* = 0.705) and Kolmogorov-Smirnov (*p* = 0.544). This finding indicates that the crystallite sizes was not affected by the exfoliation, which provided non-diffracting nanosheets [39], as well as by the treatment with H_2_O_2_.

### 3.5. FTIR Spectrometry

**Figure 4:**
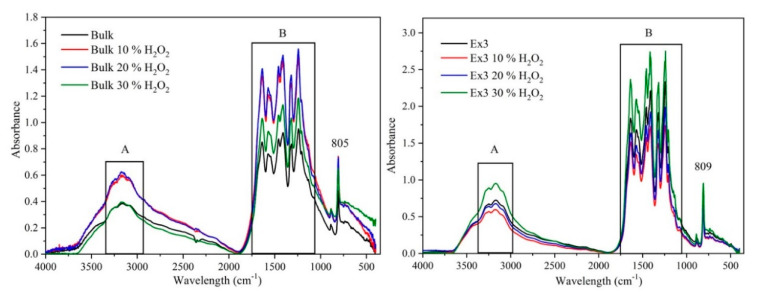
Fourier transform infrared (FTIR) spectra of bulk (**left**) and exfoliated GCN nanomaterials (**right**).

The structure of GCN nanomaterials was also studied by the FTIR spectrometry, see the spectra in Figure 4. They contained the typical bands within the areas A and B. The spectral bands in the area A are being attributed to the stretching vibrations of N-H bonds [42,43,44,45,46] and the bands in the area B are ascribed to the stretching vibrations of C=N and C-N bonds of heterocyclic rings. In addition, no C=O stretching vibrations typically located around 1700 cm^−1^ were observed likely due to their low content and/or overlap with the GCN stretching ones. Besides, the convincing presence of carbonyl groups in the FTIR spectra of the oxygen doped GCNs has not been clearly provided in the reviewed literature likely due to overlaps with the complex part B. The medium bands at 805 cm^−1^ and 809 cm^−1^ are explained by the breathing mode of triazine units. The spectral bands around 3500 cm^−1^ can be ascribed to the O-H stretching vibrations.

### 3.6. XPS Study

**Figure 5:**
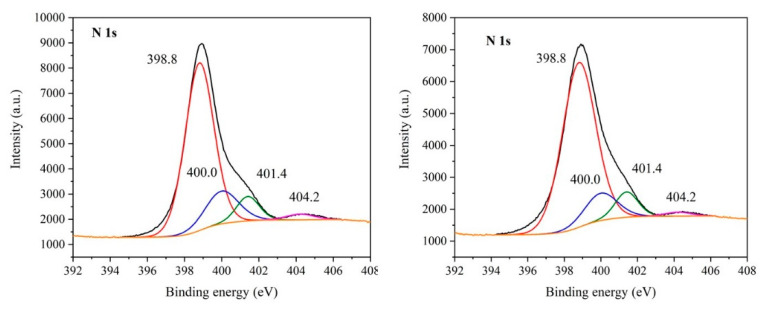
X-ray photoelectron spectrometry (XPS) spectra of N 1s of Ex3 (**left**) and Ex3 30 % H_2_O_2_ nanomaterials (**right**).

**Figure 6:**
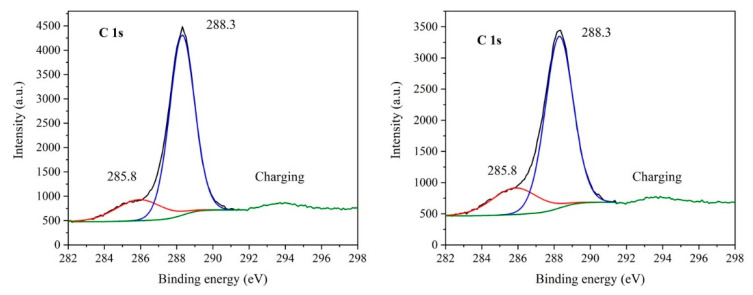
XPS spectra of C 1s of Ex3 (**left**) and Ex3 30 % H_2_O_2_ nanomaterials (**right**).

**Figure 7:**
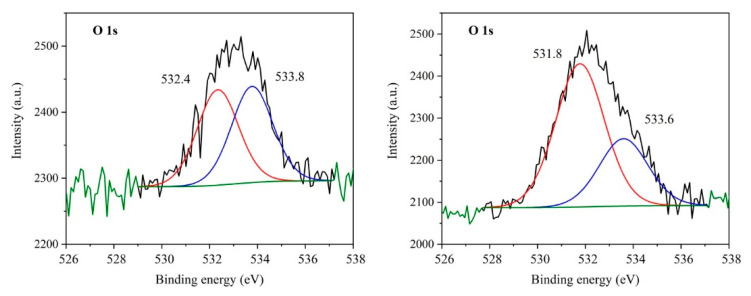
XPS spectra of O 1s of Ex3 (**left**) and Ex3 30 % H_2_O_2_ nanomaterials (**right**).

The presence of functional groups in the GCN nanomaterials was studied by XPS. Only spectra of the bulk and exfoliated (Ex3) GCNs before and after the treatment with 30% H_2_O_2_ are demonstrated in Figure 5, Figure 6 and Figure 7.

The nitrogen 1s peak (Figure 5) consisted of at least four signals at 398.8 eV, 400.0 eV, 401.4 eV and 404.2 eV [47]. The dominant part of this spectrum was the pyridinic (triazinic) NC_2_ nitrogen signal which corresponds to N atoms present at edges of the melem units. The peaks at 400.0 eV and 401.4 eV were ascribed to the NC_3_ nitrogen. The less intensive one was likely connected with bridging nitrogen atoms between melem structure cores NC_3_^B^; the more intensive one was ascribed to NC_3_^C^ nitrogen in the centres of melem units. Both signals were also attributed to the N-O groups [23,48]. The fourth peak at 404.2 eV can be ascribed to -NH_2_ or =NH groups [43,49].

The carbon 1s spectrum (Figure 6) displays two peaks at 285.5 eV and 288.3 eV of binding energies. The peak at 288.3 eV can be attributed to the CN_3_ carbon typical [50], the peak of 285.5 eV corresponds to sp^2^ hybridized carbon of C=C or CN_2_ bonds [51,52].

The spectra of oxygen are displayed in Figure 7. Both peaks were found to be composed of two components. The components at the higher binding energy at 533.8 eV and 533.6 eV can be linked with oxygen [28,53] or water [54] adsorbed on the GCN surface or with the presence of O-N bond [55,56]. The second components at 532.4 eV and 531.8 eV should be ascribed also to adsorbed water [27,28,53,57] or -OH groups [29,55,58,59] and the formation of N-C-O groups in the GCN lattice [26,28,29,32,48,60], respectively. The signal at 531.8 eV can be also attributed to the presence of C-O-C or C=O [29,53]. It is obvious that the explanation of the O bonds in GCN is difficult and the literature references are not unambiguous. In addition, the N-C-O groups often correspond to the C-OH ones.

A higher ratio of the signals at 531.8 eV and 533.6 of the Ex3 30 % H_2_O_2_ nanomaterial demonstrated in Figure 7 and the XPS results given above indicate that the following chemical reactions occurred: (i) oxidation of -OH groups to -C=O ones and (ii) formation of C-O-C groups instead of edge C-NH-C ones between two heptazine units, see Figure S3. This assumption is also supported by a little decrease of the nitrogen content after the H_2_O_2_ treatment, see Table 1.

### 3.7. SEM and TEM Analysis

**Figure 8:**
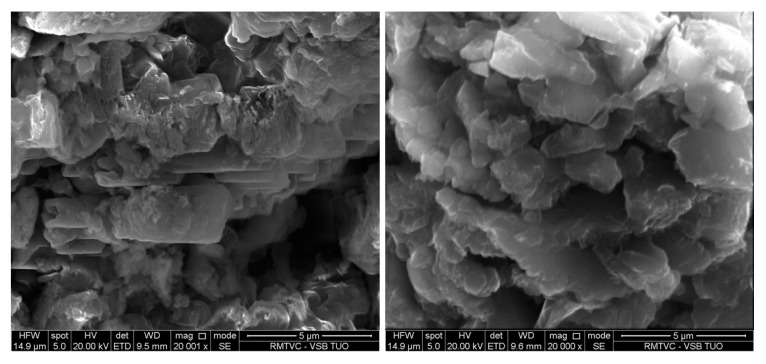
SEM images of bulk (**left**) and bulk 30 % H_2_O_2_ nanomaterials (**right**).

**Figure 9:**
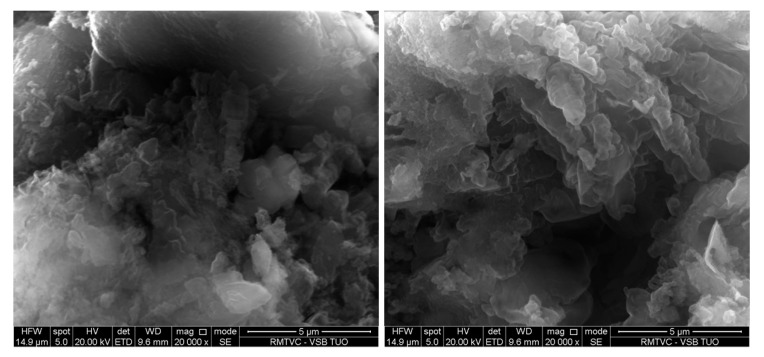
SEM images of Ex3 (**left**) and Ex3 30 % H_2_O_2_ nanomaterials (**right**).

**Figure 10:**
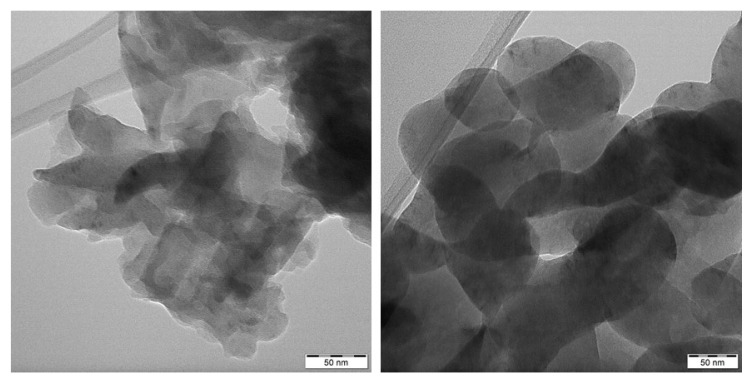
TEM images of bulk (**left**) and bulk 30 % H_2_O_2_ nanomaterials (**right**).

**Figure 11:**
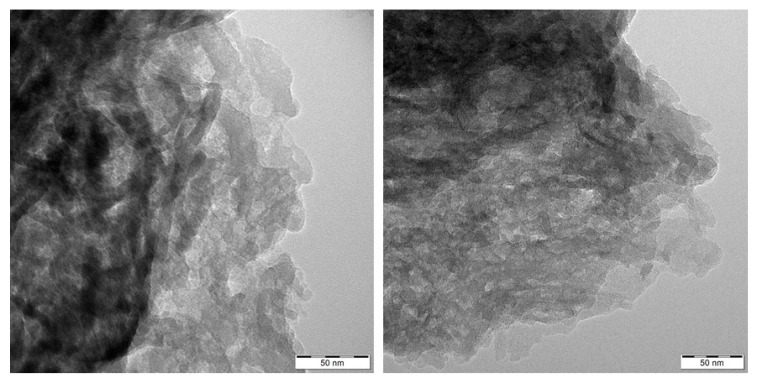
TEM images of Ex3 (**left**) and Ex3 30 % H_2_O_2_ nanomaterials (**right**).

Morphology of the GCN nanomaterials was investigated by means of SEM. Only the bulk and 3h exfoliated nanomaterials before and after the treatment are demonstrated here, in Figure 8 and Figure 9. The bulk GCNs were composed of bigger blocks than the exfoliated ones which had more disordered flake-like structures. However, the clear effect of H_2_O_2_ on the GCN morphology was not observed either for the bulk or exfoliated nanomaterials. The similar findings were obtained by the TEM analysis. Only the differences between the bulk and exfoliated materials and no effect of H_2_O_2_ were visible, see Figure 10 and Figure 11.

### 3.8. Photocatalytic Activity

The heterogeneous reaction of a compound *P* with radicals *R* formed by a photocatalytic process is being described by the Langmuir–Hinshelwood model [61] as follows

(3) $$r=-\frac{dc_{P}}{dt}=k\frac{K_{P}c_{P}}{1+K_{P}c_{P}+\sum K_{i}c_{i}}\frac{K_{R}\;c_{R}}{1+K_{R}c_{R}}$$

where *r* is the reaction rate, *k* is the kinetic constant; *K_P_*, *K_R_*, *K_i_* and *c_P_*, *c_R_*, *c_i_* are the adsorption constants and the concentrations of the remaining compound *P*, radicals *R*, and intermediates, respectively. The mechanisms of photogeneration of hydroxyls and superoxide radicals can be found anywhere in the literature, for example [62,63,64,65,66,67,68]. The widely accepted Langmuir–Hinshelwood model is based on adsorption of reacting species (*P* and *R*) on a photocatalyst´s surface. Therefore, photocatalytic reactions with the selected dyes AO7 and RhB were used for the study of the GCN surface modification with H_2_O_2_.

**Figure 12:**
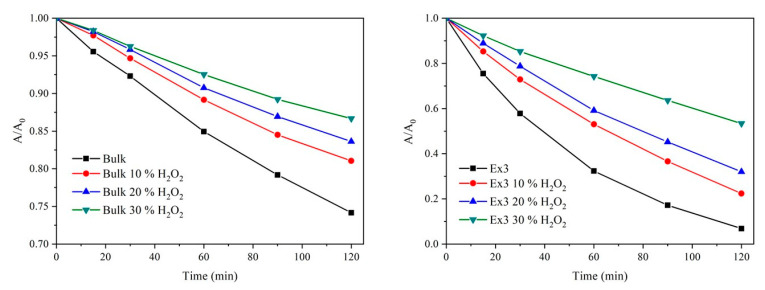
Photocatalytic degradation of AO7 in suspensions of bulk (**left**) and selected exfoliated GCN nanomaterials (**right**).

The photocatalytic degradation of AO7 in the presence of the bulk GCN and exfoliated GCN nanomaterials (Ex3) is demonstrated in Figure 12. Absorbances A for *t* = *t* and A_0_ for *t* = 0 were measured at 485 nm. The photocatalytic decomposition using the GCNs exfoliated for 1 and 2 h are demonstrated in Supplementary Materials (Figure S4). In all cases, one can see that the photocatalytic activity of the nanomaterials decreased according to the increasing concentration of H_2_O_2_ used for their treatment. Since the band gap energies were not significantly affected the possible explanation is in the lower adsorption of AO7 to the GCN nanomaterials due to their surface modifications. The AO7 molecule has a negative charge (-SO_3_^−^ group) and supposing that the dissociated -OH and/or strongly polarized -C=O groups were formed repulsive electrostatic forces between AO7 and the surfaces can be expected. The low photocatalytic activity of the bulk GCNs was explained by their low SSA.

In order to confirm these findings, the positively charged RhB was used for the next photocatalytic experiments. The oxidative degradation pathway of RhB is well known: the original red colour is changed to faint yellow which then turns colourless [69,70]. The absorption maxima of these two-colour forms correspond to 554 nm and 496 nm, respectively. The spectra of partially decomposed RhB after 60 min and 120 min shown in Figure S5 demonstrate that the original RhB molecule (red colour form, 554 nm) was primarily decomposed to a neutral fragment (yellow colour form, 496 nm) [70].

**Figure 13:**
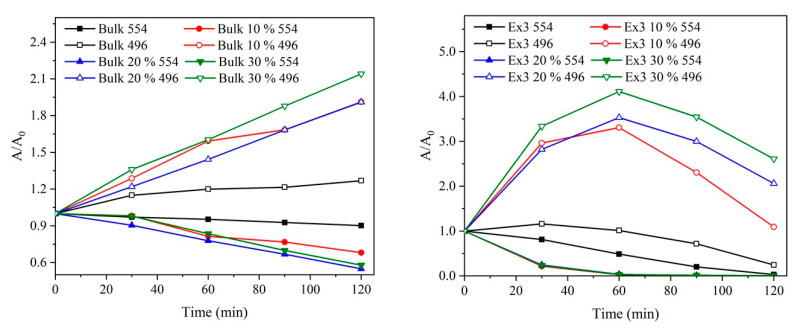
Photocatalytic degradation of RhB in suspensions of bulk (**left**) and selected exfoliated GCN nanomaterials (**right**).

Figure 13 shows the photocatalytic degradation of RhB using the bulk and exfoliated GCN nanomaterials (for 3 h) in analogy with the experiments with AO7. In case of the bulk GCN, the red-colour form was continually decomposed forming the yellow-colour one which was not decomposed at all. On the contrary, when the exfoliated GCNs were used both forms were decomposed during the photocatalytic process. The decomposition efficiency of the yellow-colour form after 120 min decreased in the sequence Ex3 > Ex3 10 % > Ex3 20 % > Ex3 30 % which is in line with the experiments with AO7 and thus confirms an idea of the GCN surface modification with the negatively charged groups.

### 3.9. Measurement of Zeta Potentials

**Figure 14:**
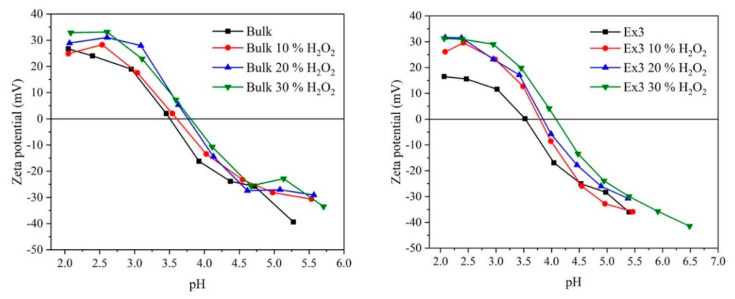
Zeta potentials of bulk (**left**) and selected exfoliated GCN nanomaterials (**right**) depending on pH.

The surface charge of the GCN nanomaterials is supposed to be determined in part by pH of their aqueous suspensions. Therefore, their zeta potentials were measured in dependence on pH demonstrated in Figure 14 for us to find how surfaces of the GCN nanomaterials were altered by the treatment with H_2_O_2_. The H^+^ and OH^−^ ions are potential-determining ions due to reactions of H^+^ ions with -NH_2,_ edge >NH, =N- and -OH groups forming -NH_3_^+^, >NH_2_^+^, =NH^+^- and -OH_2_^+^ ones [71,72], and with OH^−^ ions forming -O^−^ species.

From the measured data points of zero charges (p.z.c.) were evaluated, see Table 4. One can see that the zeta potentials increased after the treatment. The exfoliation likely facilitated the access of H^+^ ions to amino groups providing the positive charge but, on the other hand, caused the cracking of GCN planes which formed hydroxyl groups providing the negative charge at higher pH. The zeta potential increase is the most remarkable in case of Ex3 when p.z.c. 3.51 of the bulk GCN increased to 4.08 of the one treated in 30% H_2_O_2_. The p.z.c. values agree with those published in the literature, e.g., [67,71,73].

As mentioned above, the zeta potential increase can be explained by the less negative surface charge caused by the partial oxidation of hydroxyl groups to carbonyl ones. Then, the lower amount of dissociated -OH species should require the lower amount of H^+^ ions necessary for their charge neutralization. Supposing that oxygen was present in the N-C-O groups, the existence of C-O^−^ species suggested by Yang et al. [31] could decrease the zeta potential which was not observed. It can be claimed that the zeta potential measurements were in agreement with the results of the photocatalysis and XPS and confirmed the GCN surface modification.

In addition, the aqueous suspensions of the bulk GCN nanomaterials (0.03 g in 100 mL) were titrated with HCl (0.025 mol L^−1^) and NaOH (0.025 mol L^−1^) solutions. The starting pH of their aqueous suspensions was 5.6–5.9 although pH of distilled water was 6.9. It means that the GCN nanomaterials released hydrogen ions into the suspensions as a result of dissociation of weak acidic groups. The titration curves in Figure S6 demonstrate the big increase of pH in case of the treated bulk GCN nanomaterials likely due to the oxidation of weak acidic groups (-OH and >NH_2_^+^) which were not able to neutralize added hydroxide. Hydroxide ions can be also exchanged with other anions in the GCN structure [74] and consumed by the neutralization of protonated nitrogen and amino groups of GCN [72].

## 4. Conclusions

Bulk GCN was synthetized from melamine in air and then thermally exfoliated in air as well. The bulk and exfoliated GCN nanomaterials were treated in 10–30% aqueous solutions of hydrogen peroxide. The treatment of GCN led to the doping with oxygen as found by EA. Hydrogen peroxide was supposed to partially oxidize -OH groups to C=O ones and to form the edge C-O-C groups instead of C-NH-N ones which was recognized by the reddish colour of the treated nanomaterials and the results of spectral analyses (UV-Vis DR, PL, and FTIR spectroscopy, XPS).

The surface modification was documented by the different photocatalytic activities tested by the degradation of AO7 and RhB, the changed zeta-potentials and neutralization titration curves of the treated GCNs. The H_2_O_2_ treatment did not affect the nanomaterial structures which was observed by the XRD, SEM, TEM, and SSA measurements.

It can be concluded that hydrogen peroxide is capable of doping the GCN nanomaterials with oxygen which leads to their modification associated with the changes of their light absorption and surface adsorption properties. This surface modification allows us to prepared new photocatalysts for the degradation of various compounds in gaseous and liquid phases. The future research will be focused on degradation of various organic and inorganic environmental pollutants.

## Figures and Tables

**Table 1:** Elemental analysis of graphitic carbon nitride (GCN) nanomaterials.

Nanomaterial	C (wt.%)	H (wt.%)	N (wt.%)	C/N	O (wt.%)
Bulk	34.93	1.72	61.47	0.568	1.88
Bulk 10 % H_2_O_2_	34.11	1.87	60.25	0.566	3.77
Bulk 20 % H_2_O_2_	33.21	1.92	58.86	0.564	6.01
Bulk 30 % H_2_O_2_	34.25	1.60	60.53	0.566	3.62
Ex1	34.14	1.79	60.30	0.566	3.77
Ex1 10 % H_2_O_2_	33.24	2.05	59.25	0.561	5.46
Ex1 20 % H_2_O_2_	34.13	1.73	60.54	0.564	3.60
Ex1 30 % H_2_O_2_	33.76	1.77	59.65	0.566	4.82
Ex2	34.22	1.79	60.57	0.565	3.42
Ex2 10 % H_2_O_2_	33.16	2.29	59.11	0.561	5.44
Ex2 20 % H_2_O_2_	33.59	1.86	59.50	0.565	5.05
Ex2 30 % H_2_O_2_	33.56	1.84	59.46	0.564	5.14
Ex3	33.85	1.83	59.97	0.564	4.35
Ex3 10 % H_2_O_2_	32.97	2.07	58.90	0.560	6.06
Ex3 20 % H_2_O_2_	33.03	1.85	58.60	0.564	6.52
Ex3 30 % H_2_O_2_	33.76	1.82	59.76	0.565	4.66

**Table 2:** Band gap energy and specific surface area values of GCN nanomaterials.

Nanomaterial	E_g_ (eV)	SSA (m^2^ g^−1^)	Nanomaterial	E_g_ (eV)	SSA (m^2^ g^−1^)
Bulk	2.65	12	Ex2	2.73	93
Bulk 10 % H_2_O_2_	2.59	11	Ex2 10 % H_2_O_2_	2.61	107
Bulk 20 % H_2_O_2_	2.59	9	Ex2 20 % H_2_O_2_	2.59	113
Bulk 30 % H_2_O_2_	2.59	14	Ex2 30 % H_2_O_2_	2.57	101
Ex1	2.71	58	Ex3	2.75	143
Ex1 10 % H_2_O_2_	2.60	83	Ex3 10 % H_2_O_2_	2.65	117
Ex1 20 % H_2_O_2_	2.60	93	Ex3 20 % H_2_O_2_	2.64	116
Ex1 30 % H_2_O_2_	2.56	98	Ex3 30 % H_2_O_2_	2.61	104

**Table 3:** XRD characteristics of GCN nanomaterials.

Nanomaterial	2 Theta (deg)	FWHM (deg)	L(002) (nm)	*d*(002) (nm)
Bulk	31.84	1.31	7.0	0.326
Bulk 10 % H_2_O_2_	32.11	1.32	7.0	0.323
Bulk 20 % H_2_O_2_	32.10	1.55	6.0	0.324
Bulk 30 % H_2_O_2_	32.60	1.37	6.7	0.319
Ex1	31.88	1.32	7.0	0.326
Ex1 10 % H_2_O_2_	31.99	1.05	8.8	0.325
Ex1 20 % H_2_O_2_	32.19	1.25	7.4	0.323
Ex1 30 % H_2_O_2_	32.60	1.21	7.6	0.319
Ex2	31.84	1.36	6.8	0.326
Ex2 10 % H_2_O_2_	32.05	1.01	9.1	0.324
Ex2 20 % H_2_O_2_	32.25	1.25	7.4	0.322
Ex2 30 % H_2_O_2_	32.62	1.13	8.2	0.319
Ex3	31.88	1.29	7.2	0.326
Ex3 10 % H_2_O_2_	32.07	1.01	9.1	0.324
Ex3 20 % H_2_O_2_	32.09	1.23	7.5	0.324
Ex3 30 % H_2_O_2_	32.63	1.16	8.0	0.318

Note: The 2 Theta values correspond to the (002) diffractions.

**Table 4:** Points of zero charges of bulk and selected exfoliated GCN nanomaterials.

Nanomaterial	p.z.c.	Nanomaterial	p.z.c.
Bulk	3.51	Ex3	3.53
Bulk 10 % H_2_O_2_	3.60	Ex3 10 % H_2_O_2_	3.79
Bulk 20 % H_2_O_2_	3.76	Ex3 20 % H_2_O_2_	3.85
Bulk 30 % H_2_O_2_	3.80	Ex3 30 % H_2_O_2_	4.08

## Supplementary Materials

The following are available online at https://www.mdpi.com/2079-4991/10/9/1747/s1, Figure S1: Images of bulk GCN nanomaterials, Figure S2: Images of exfoliated GCN nanomaterials, Figure S3: Melon structure with supposed position of oxygen, Figure S4: Photocatalytic degradation of AO7 in suspensions of exfoliated GCN nanomaterials, Figure S5: Spectra of RhB after 60 min (left) and after 120 min of the photocatalytic degradation with exfoliated GCN, Figure S6: Titration curves of bulk GCN nanomaterials.
