# Supplementary material for: Modification of Graphitic Carbon Nitride with Hydrogen Peroxide
Source: Nanomaterials (Basel). 2020 Sep 3;10(9):1747. doi: 10.3390/nano10091747 (PMC7559342; doi:10.3390/nano10091747)
Supplement: Supplementary file 1 [file nanomaterials-10-01747-s001.pdf]

nanomaterials

Supplementary materials

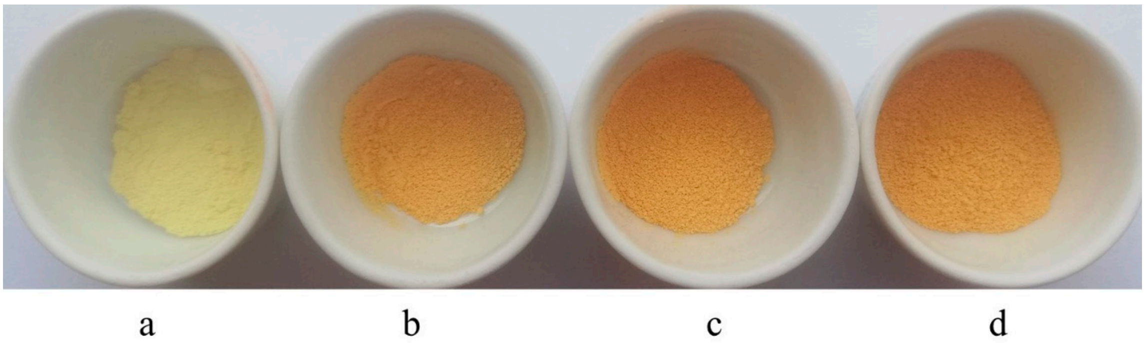

Figure S1. Images of bulk CN nanomaterials. (**a**) Bulk, (**b**) Bulk 10 % H_2O_2, (**c**) Bulk 20 % H_2O_2, (**d**) Bulk 30 % H_2O_2.

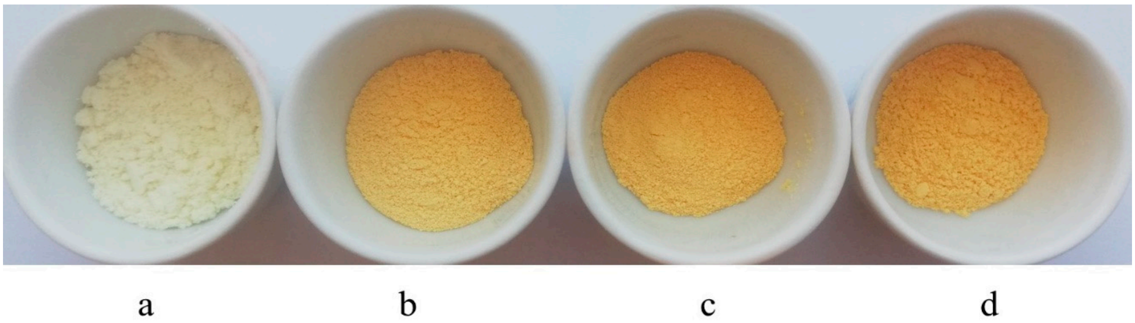

Figure S2. Images of exfoliated CN nanomaterials. (**a**) Ex3, (**b**) Ex3 10 % H_2O_2, (**c**) Ex3 20 % H_2O_2, (**d**) Ex3 30 % H_2O_2.

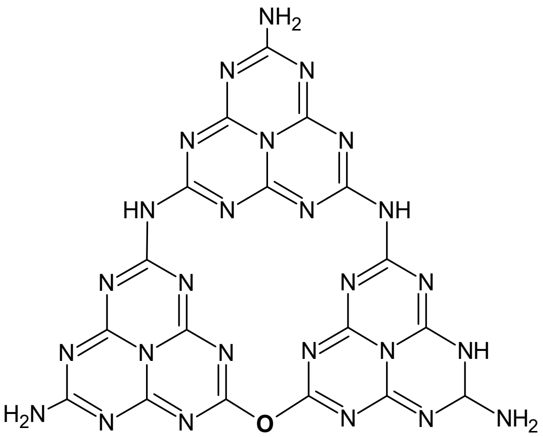

Figure S3. Melon structure with supposed position of oxygen.

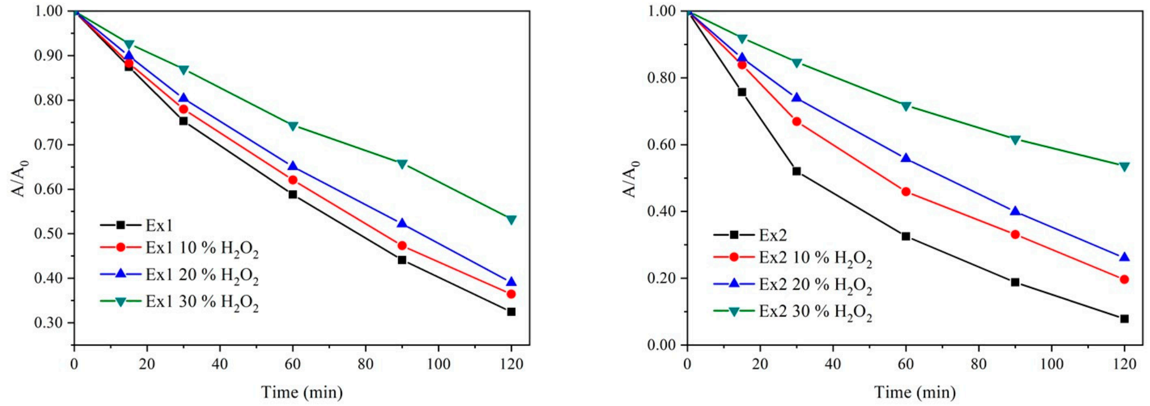

Figure S4. Photocatalytic degradation of AO7 in suspensions of exfoliated CN nanomaterials.

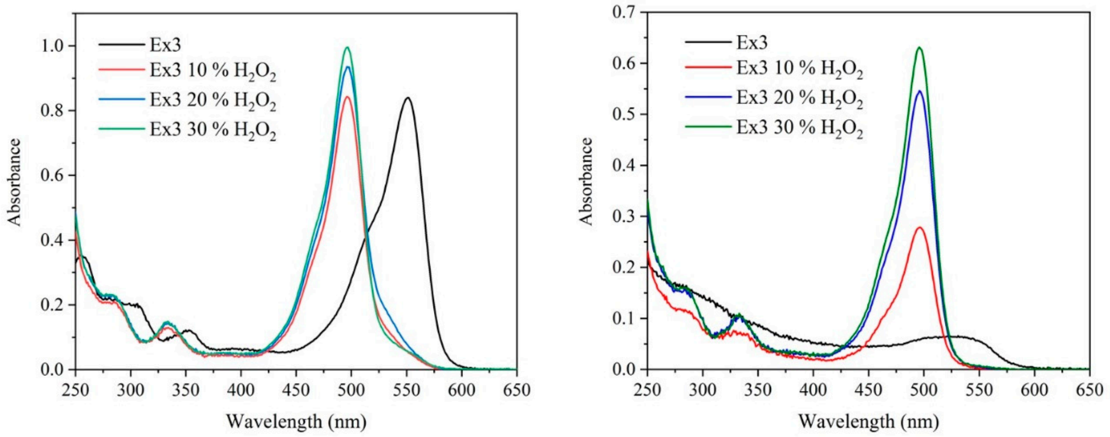

Figure S5. Spectra of RhB after 60 min (**left**) and after 120 min of the photocatalytic degradation with exfoliated CN.

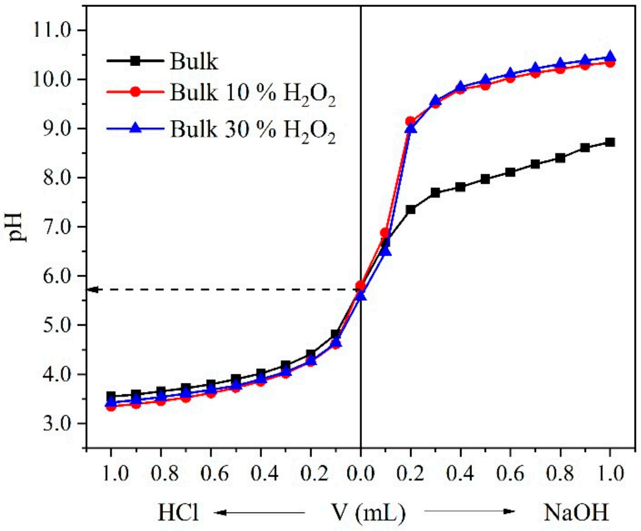

Figure S6 Titration curves of bulk CN nanomaterials.
